# Surrounding species diversity improves subtropical seedlings’ carbon dynamics

**DOI:** 10.1002/ece3.4225

**Published:** 2018-06-22

**Authors:** Yann Salmon, Xuefei Li, Bo Yang, Keping Ma, Rolf T. W. Siegwolf, Bernhard Schmid

**Affiliations:** ^1^ Department of Evolutionary Biology and Environmental Studies University of Zurich Zurich Switzerland; ^2^ School of Geosciences University of Edinburgh Edinburgh UK; ^3^ Institute for Atmospheric and Earth System Research/Physics Faculty of Science University of Helsinki Helsinki Finland; ^4^ Institute for Atmospheric and Earth System Research/Forest Sciences Faculty of Agriculture and Forestry University of Helsinki Helsinki Finland; ^5^ Key Laboratory of Speciality Resources Biodiversity of Jiangxi Province Jingdezhen University Jingdezhen Jiangxi China; ^6^ State Key Laboratory of Environment and Vegetation Change Institute of Botany Chinese Academy of Sciences Xiangshan, Beijing China; ^7^ Lab for Atmospheric Chemistry, Ecosystem Fluxes and Stable Isotope Research Paul Scherrer Institute Villigen Switzerland

**Keywords:** ^13^C, ^18^O, biodiversity, competition, photosynthesis, respiration, stable isotope, water

## Abstract

Increasing biodiversity has been linked to higher primary productivity in terrestrial ecosystems. However, the underlying ecophysiological mechanisms remain poorly understood. We investigated the effects of surrounding species richness (monoculture, two‐ and four‐species mixtures) on the ecophysiology of *Lithocarpus glaber* seedlings in experimental plots in subtropical China. A natural rain event isotopically labelled both the water uptaken by the *L. glaber* seedlings and the carbon in new photoassimilates through changes of photosynthetic discrimination. We followed the labelled carbon (C) and oxygen (O) in the plant–soil–atmosphere continuum. We measured gas‐exchange variables (C assimilation, transpiration and above‐ and belowground respiration) and δ^13^C in leaf biomass, phloem, soil microbial biomass, leaf‐ and soil‐respired CO_2_ as well as δ^18^O in leaf and xylem water. The ^13^C signal in phloem and respired CO_2_ in *L. glaber* in monoculture lagged behind those in species mixture, showing a slower transport of new photoassimilates to and through the phloem in monoculture. Furthermore, leaf‐water ^18^O enrichment above the xylem water in *L. glaber* increased after the rain in lower diversity plots suggesting a lower ability to compensate for increased transpiration. *Lithocarpus glaber* in monoculture showed higher C assimilation rate and water‐use efficiency. However, these increased C resources did not translate in higher growth of *L. glaber* in monoculture suggesting the existence of larger nongrowth‐related C sinks in monoculture. These ecophysiological responses of *L. glaber*, in agreement with current understanding of phloem transport are consistent with a stronger competition for water resources in monoculture than in species mixtures. Therefore, increasing species diversity in the close vicinity of the studied plants appears to alleviate physiological stress induced by water competition and to counterbalance the negative effects of interspecific competition on assimilation rates for *L. glaber* by allowing a higher fraction of the C assimilated to be allocated to growth in species mixture than in monoculture.

## 1 INTRODUCTION

Plant species diversity has been shown to positively impact ecosystem primary production (Balvanera et al., 2006). However, the effects of biodiversity in forest ecosystems have only started to be investigated (e.g., Morin, Fahse, de Mazancourt, Scherer‐Lorenzen, & Bugmann, 2014; Morin, Fahse, Scherer‐Lorenzen, & Bugmann, 2011; Potvin & Gotelli, 2008); and general conclusions are still lacking, despite the major carbon (C) sink that forests represent (Pan et al., 2011). This lack of knowledge leaves us with a limited understanding of the true extent of local biodiversity effects on ecosystem functioning and its role in carbon sequestration, especially in highly productive regions such as the tropics and subtropics, which account for a large—albeit threatened (Brienen et al., 2015)— fraction of the terrestrial C sink (Bonan, 2008; Phillips & Lewis, 2014).

Hence, given that primary productivity reflects the balance between photosynthetic C assimilation and respiratory C loss and is a result of the allocation of recently assimilated C within the plant–soil continuum, the positive effects of plant diversity on primary productivity and biomass accumulation in ecosystems should result from changes of these three physiological processes—namely, assimilation, respiration and C allocation—at the ecosystem level. Furthermore, these processes are of vital importance for the dynamics of C pools in terrestrial ecosystems (Kuzyakov & Gavrichkova, 2010). Understanding their control is crucial to model and estimate C budgets in terrestrial ecosystems under changing environmental conditions (Litton, Raich, & Ryan, 2007). Despite its potentially large impact on C budgets in ecosystem, little is known about the effect of species richness on C dynamics and its regulatory processes at the level of whole‐plant physiology. One of the few studies addressing the effects of biodiversity on C dynamics in terrestrial ecosystems showed that in temperate grassland increasing species diversity leads to higher C assimilation at the community level (De Boeck et al., 2007) confirming the importance of physiological adaptations of individual plants to their surrounding diversity as component of the biotic environment.

Also complex and not fully understood to date (Savage et al., 2016), phloem likely plays a key role in allowing plants to adjust C allocation in response to their environment, as it represents the main pathway for new assimilate transfer from C sources to C sinks. The most widely accepted conceptual framework of phloem transport is the Münch hypothesis: C transport from source (leaves) to sink organs (heterotrophic tissues, rhizosphere, etc.) in phloem is driven by water flow in xylem and an osmotic gradient. A hydrostatic pressure difference between C sources and sinks results from the loading of assimilates near the C sources, which increases osmotic pressure and consequently drives water out of the xylem and into the phloem, and from sugar unloading near the C sinks, which has the opposite effect (Van Bel, 2003). When water availability decreases, competition for water between transpiration and phloem transport increases, resulting in more viscous sap that moves slower (Hölttä, Vesala, Sevanto, Perämäki, & Nikinmaa, 2005; Lacointe & Minchin, 2008). Therefore, to understand C dynamics in the plant–soil–atmosphere continuum, potential adjustments of phloem‐C transport in response to biotic and abiotic environmental variation should be studied.

Stable isotopes have become a widely‐used tool to trace C fluxes through ecosystems and gain information about the underlying physiological processes controlling these fluxes (Brüggemann et al., 2011; Dawson, Mambelli, Plamboeck, Templer, & Tu, 2002; Epron et al., 2012). The isotope signature (δ^13^C) of newly assimilated C by a plant leaf is affected by photosynthetic discrimination (∆_*i*_) resulting from the physiological response of the plant leaf (regarding C assimilation and water loss) to environmental conditions (Farquhar, Ehleringer, & Hubick, 1989). Thus, newly assimilated C can be traced in the plant–soil–atmosphere continuum. This method has often been used to understand the impact of environmental variables on the C cycle (see reviews by Brüggemann et al., 2011; Kuzyakov & Gavrichkova, 2010; Mencuccini & Hölttä, 2010), even though recent studies have highlighted the need to deal with confounding effects, which might modify the isotope signal between assimilation and respiration. Such effects include diel variations of δ^13^C in respired CO_2_, postphotosynthetic and respiration fractionation and damping of the ^13^C signal as it is transferred below ground (Gessler, Tcherkez, Peuke, Ghashghaie, & Farquhar, 2008; Kodama et al., 2008; Werner & Gessler, 2011). Furthermore, in recent years, biological controls (ontogeny, physiological adaptation to biotic and abiotic environment) have emerged as additional important drivers of short‐term C dynamics and its isotope signature (e.g., Bathellier et al., 2008; Ghashghaie & Badeck, 2014; Ghashghaie et al., 2015; Salmon, Barnard, & Buchmann, 2011, 2014; Salmon, Buchmann, & Barnard, 2016).

Because phloem transport and consequently the ability of plants to allocate C from leaves to sink organs depend strongly on the balance between available C and available water, it is expected that water resources should influence plant growth and carbon balance. Stable isotope ratios such as δ^18^O in xylem water can provide information about the water source for the plant (Dawson et al., 2002; Ehleringer & Dawson, 1992), while δ^18^O in leaf water—although more complex to understand—reflects water exchange between the plant and the environment (e.g., Simonin et al., 2013). Thus, stable isotopes provide a powerful tool to understand short‐term C dynamics and its internal plant physiological and external abiotic and biotic drivers.

To improve our understanding of the effects of biodiversity on plant physiology and short‐term C dynamics in subtropical forest ecosystems, we set up small model communities of 16 young trees of 1, 2 or 4 species in the framework of the so‐called BEF‐China project (Bruelheide et al., 2014). In these model communities, we studied short‐term fluxes of C and water in the target species *Lithocarpus glaber* using stable C and O‐isotopes. A rain event was used to naturally label C and water‐O and trace assimilated C and absorbed water‐O in the days following that event. We hypothesized that (a) the species richness of the model communities could positively affect short‐term C and water fluxes in the target species due to reduced intraspecific competition for water; and (b) the increased short‐term fluxes of C and water could result in positive biodiversity effects on primary productivity of *L. glaber* in the model communities.

## 2 MATERIALS AND METHODS

### 2.1 Study site and experimental design

The study was carried out in Jiangxi Province in southeast China (N29°06.293 E117°55.286). The climate at the study site is subtropical with a mean annual temperature or 17.4°C, a mean annual precipitation of 1,635 mm and a distinct seasonality of a hot‐humid season from May to July and a cool‐dry season from October to March (Scholten et al., 2017). Soils in the region are mainly Cambisols with Acrisols and Ferrasols in the lower regions (Lang et al., 2014). The regional subtropical forest is characterized by a high diversity of woody plant species with evergreen species dominating in terms of number of individuals (Bruelheide et al., 2011).

Our experiment was part of the same tree diversity experiment as described by Lang et al. (2014) and was used as a pilot experiment for the so‐called BEF‐China project (Bruelheide et al., 2014; Schmid, Baruffol, Wang, & Niklaus, 2017). Our experiment was set up on a former agricultural field, which was plowed and harrowed in March 2009. Prior to the experiment, rice, rape and vegetables were grown on the site in a double‐cropping system. Following plowing, the field was divided into four blocks of 1,975 m^2^. Each block was divided into 1‐m^2^ sized plots separated by 20 cm deep and 75 cm wide vegetation‐free spaces (Schmid et al., 2017). Channels were dug around blocks and connected to trenches allowing drainage of excess rain water.

**Figure 1:**
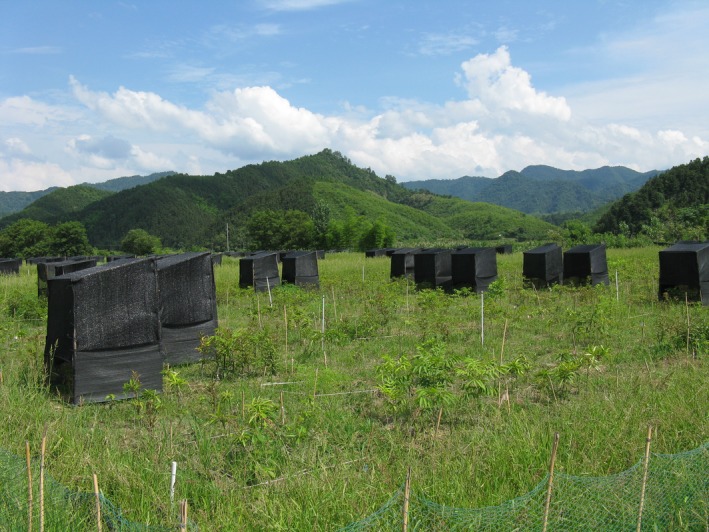
Photograph of the experimental sites. The 16 plants plots are limited by sticks and rope. The shade house was not part of the present experiment

In the present study a subset of plots from the experiment was used (Figure 1). These plots were planted with *L. glaber* (Thunb.) Nakai in monoculture, two‐species mixtures of *L. glaber* with *Castanopsis sclerophylla* (Lindl. & Paxton) Schottky, *Cyclobalanopsis myrsinaefolia* Blume or *Sapindus mukorossi* Gaertn and finally the mixture of these four species, leading to a total of five species compositions of experimental communities. Each of the five species compositions was represented by one randomly placed replicate in each of the four blocks, leading to a total of 20 experimental communities. Each plot was planted with 16 individuals. The young trees had been previously grown from seeds in a tree nursery for 6–12 months until they had reached a planting size of about 30 cm.

### 2.2 Plant material

*Lithocarpus glaber* was used as a “phytometer” (Mwangi et al., 2007), that is, standardized plant material (same species, age and size of individuals), in our experiment. Using such a phytometer we could exclude ontogenetic or species‐specific variation in measured isotopic values (e.g., Bathellier et al., 2008; Ghashghaie & Badeck, 2014; Priault, Wegener, & Werner, 2009; Salmon et al., 2011, 2016) and focus on the effects of the species diversity of the experimental communities on the target species.

A day preceding and 3 days following a rain event (38 mm), measurements were taken daily on three randomly selected individuals in three plots (i.e., one individual per plot) at each of the three levels of diversity, that is, in monocultures, two‐species mixtures and four‐species mixtures. For the two‐species mixtures, all species combinations were sampled. Plants were too small to shade each other (no light competition), but rooting systems of different individuals overlapped below ground as verified by excavation at the end of the experiment.

### 2.3 Meteorological data

**Figure 2:**
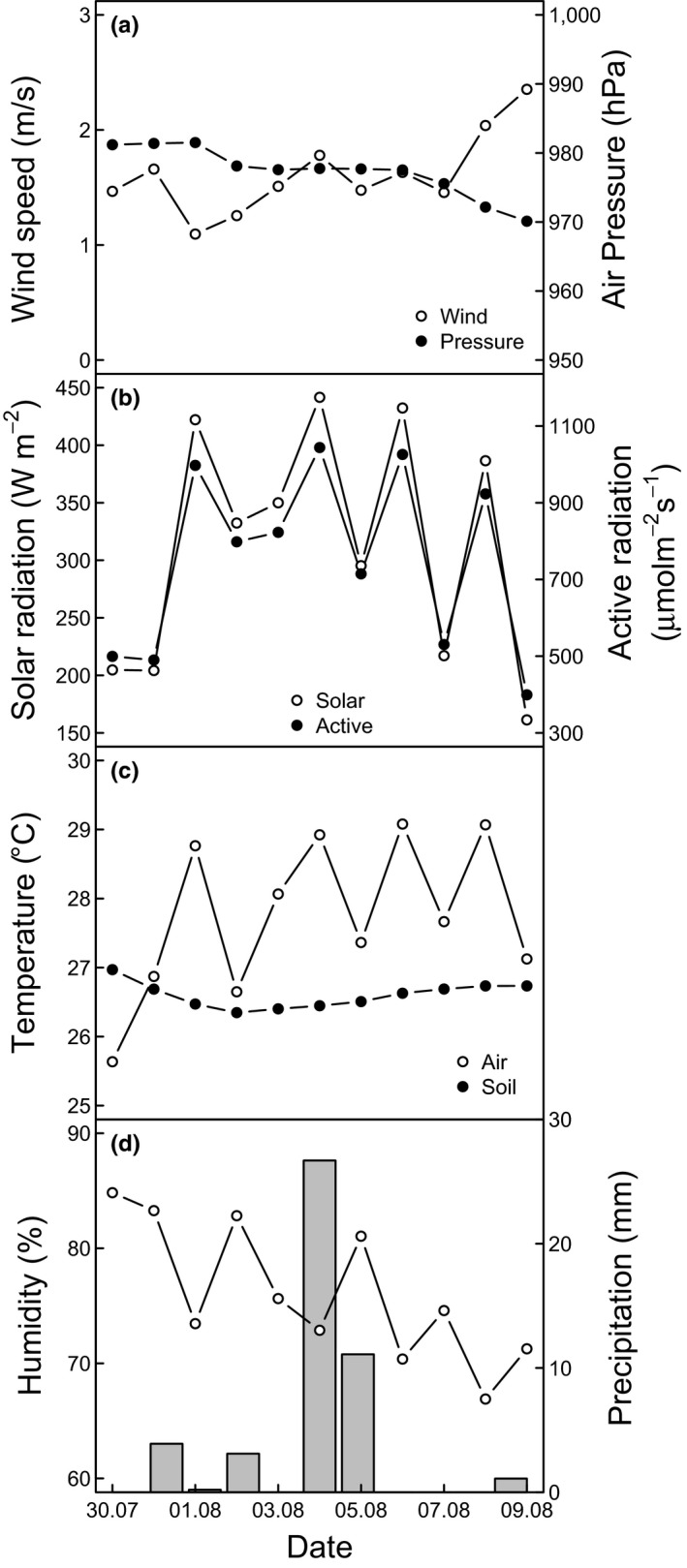
Daily meteorological average values before, during and after the experiment: Panel (a) daily wind speed (m/s), daily atmospheric pressure (hPa); Panel (b) day time (W/m^2^) average solar radiation and photosynthetically active radiation (PAR, μmol m^−2 ^s^−1^); Panel (c) daily average air and soil temperature at 20 cm soil depth (°C); Panel (d) daily averaged air humidity (RH, %) and total daily precipitation (mm). Measurements started on the 4th of August (later referred as day 1) before the rain event (night between 4th and 5th of August) and were continued until the 7th of August

A meteorological station EcoTech Meteostation (EcoTech, Bonn Fon, Germany) was installed on the BEF‐China main site (Bruelheide et al., 2014), about 2 km from the present experiment. Meteorological data were recorded continuously on an ecoTech enviLog Datalogger (EcoTech), equipped with Vaisala Weather Transmitter WXT520 (Vaisala Oyj, Helsinki, Finland), precipitation gauge 2153/3 (Ecotech), Silicon Pyranometer SP Lite2 (Kipp & Zonen, Delft, The Netherlands), PAR Quantum Sensor PQS1 (Kipp & Zonen) and EcoTech soil‐temperature sensors (EcoTech) at depths of 20, 50 and 80 cm. All meteorological data were averaged over a 5‐min interval before storage. Daily averages of meteorological data are presented in Figure 2.

### 2.4 Ecophysiological and CO_2_ efflux measurements

The following ecophysiological variables (see Table 1 for the list of measured variables) were measured on fully expanded leaves of three plants of the phytometer species in three plots (i.e., one plant per plot) per diversity level (monoculture, two‐species mixture and four‐species mixture) and for all three different species compositions of diversity level 2 (i.e., *L. glaber* with either *C. sclerophylla*, or *C. myrsinaefolia* or *S. mukorossi*) and every day between 8 a.m. and 11 a.m. and between 3 p.m. and 6 p.m. (thus minimizing differences due to measurements time, for example, the midday photosynthetic depression): transpiration rate (*E*), stomatal conductance of leaves to H_2_O (*g*_*s*_), CO_2_ assimilation rate (*A*). Due to the impossibility to access the site at night time, a proxy for leaf dark respiration rate (*r*_dark_) was measured in the morning on leaves kept in the dark (covered with dark plastic bags the evening before) to avoid light‐enhanced dark respiration (Barbour, McDowell, Tcherkez, Bickford, & Hanson, 2007). A similar method was used to sample CO_2_ respired by leaves for isotope measurements (see below). Five measurements were averaged per plant. Measurements were carried out under standardized conditions with a portable photosynthesis system (Li‐6400, Li‐Cor Inc., Lincoln, NE, USA). Light measurement conditions were controlled using a light source (6400‐02B, Li‐Cor Inc.) and mirrored daily light intensity at 1,000 μmol m^−2^ s^−1^. Ambient CO_2_ concentration and relative humidity (RH) in the Li‐6400 cuvette were used during gas‐exchange measurements and remained mostly stable for the duration of the measurements (average ± 1 *SD* for CO_2_: 394 ± 24 ppm and for RH: 69 ± 9%). Leaves were kept in the chamber until gas‐exchange variables reached a steady state. Leaves that had been used for leaf gas‐exchange measurements were then cut 1 cm above the ground and leaf area was measured with a portable area meter (Li‐3000C, Li‐Cor Inc.) before drying (48 hr at 60°C) and weighing.

Soil CO_2_ efflux rate (also referred to as soil respiration to simplify the syntax) was measured daily within 10 cm of each phytometer plant that was used for aboveground measurements. It should be noted that although the present protocol aims at minimizing it, contributions from other species to the measured efflux cannot be excluded. A custom‐made PVC chamber (4 cm high, 7 cm long, 5 cm wide) equipped with a septum for gas sampling was tightly placed on cleaned soil using a large closed cell foam ring to seal it, and connected to a CO_2_/H_2_O gas analyser (Li‐840, Li‐Cor Inc.). The absence of leaks was tested prior to measurements by blowing around the chamber and monitoring the CO_2_ response in the chamber. Once the system was airtight, soil CO_2_ efflux rate was calculated over 1 min of linearly increasing CO_2_ concentrations in the chamber.

**Table 1:** List of the variables used in the manuscript with their respective symbol and their biological meaning

Variable	Symbol	Meaning for plant function
Ecophysiological variables		
CO_2_ assimilation	*A*_*N*_	Other things being equal, *A*_*N*_ should increase after the rain. Higher assimilation means more resources for growth, secondary metabolism, etc
Stomatal conductance	*g*_*s*_	Regulate C gain versus water loss between the inside of the leaf and the atmosphere. Usually increase as the stomata open with increasing water availability
Transpiration	*E*	Water loss when stomata are open to allow photosynthesis. Usually increase with increasing water availability
Leaf dark respiration	*r*_dark_	Represent the energy spend by the trees leaves. It can be the results of growth, production of secondary metabolites, and maintenance of basic physiological need (Usually a very minor fraction of the total respiration in nonstressed plants)
Soil CO_2_ efflux	*r*_s_	The sum of autotrophic (roots) and heterotrophic (mostly microbial) respiration plus changes in diffusion processes of CO_2_ from the soil internal atmosphere to the soil surface
Derived photosynthetic C‐isotope discrimination (simple model)	Δ_*i*_	Changes in the ratio of ^13^C‐ over ^12^C‐CO_2_ fixed during photosynthesis. Decrease with water stress and stomatal closure. Changes in Δ_*i*_ allow to label newly assimilated C
Water‐use efficiency	WUE	Trade‐off between water lose and carbon gain, increases with water limitation
^13^C variables		
δ^13^C value of phloem organic matter	δ^13^C_phloem_	Changes according to the isotopic signature of the new photoassimilates and how fast they are transported in the phloem from the leaves and out of the phloem from the C sink
δ^13^C value of leaf‐respired CO_2_	δ^13^C_Rleaf_	Changes according to the δ^13^C of the C respired, which depends on the δ^13^C of new photoassimilates in nonstressed plants and on metabolic processes responsible the changes in Δ_Rphloem‐leaf_ (see Supporting Information)
δ^13^C value of soil‐respired CO_2_	δ^13^C_Rsoil_	Changes according to the isotopic signature of the C transported belowground by the phloem and by changes in C pool used by the roots and soil microbial community to fuel their respiration (this influence Δ_Rphloem‐soil_, see Supporting Information)
^18^O variables		
δ^18^O value of xylem sap	δ^18^O_xylem‐water_	δ^18^O of the source water (i.e., water taken up by the roots). Hence, changes in δ^18^O_xylem‐water_ mean changes in the water used by the plants
δ^18^O value of leaf water	δ^18^O_leaf‐water_	δ^18^O of the water in the leaf, it is affected by δ^18^O of water source and by fractionation happening during the transpiration which tend to enrich the leaf water in ^18^O
Leaf water enrichment in ^18^O relative to that of the source water	Δ^18^O_leaf‐water_	Changes in Δ^18^O_leaf‐water_ reflects the effects of transpirations on δ^18^O_leaf‐water_. It increases with increasing transpiration

### 2.5 Plant and soil sample collection for isotopic analysis

Leaves, roots, soil and phloem organic matter of or near the phytometer plants were sampled at the end of the measurement day. A first soil core (5‐cm diameter core, 10 cm deep) was taken for bulk‐soil δ^13^C measurements after manually removing roots. A subsample of the mixed samples was taken for isotopic analysis (see below). A second soil core (5 cm diameter, 10 cm deep) was taken for microbial biomass δ^13^C measurements (see Supporting Information Appendices S1 and S2). To minimize the contribution of the other species to bulk soil and microbial biomass δ^13^C measurements, both soil cores were taken with the edge of the core within 2 cm of the phytometers stem. Roots of the phytometer plants were separated from a third soil core by wet sieving. However, the amount of root biomass available proved too small for isotopic analyses. Leaf and soil samples for isotope‐composition analysis were dried (48 hr at 60°C) and finely ground (further details are provided in Supporting Information Appendices S1 and S2). Gravimetric soil water content was calculated after drying approximately 10 g of soil at 105°C. Bulk phloem organic matter was collected using an exudation method (Gessler, Keitel, Nahm, & Rennenberg, 2004). Briefly, for each replicate, one 5‐cm twig was cut, rinsed with ultrapure water and carefully dabbed. Then, twigs were inserted in a tube filled with 2 ml of 0.15 M polyphosphate buffer at pH 7.5, sealed with parafilm^®^ and placed in the dark (100% humidity, 4°C). After 5 hr, 1.5 ml of solution were collected, lyophilized and used for C‐isotope‐composition analysis (see below). Given the very small amount of phloem sap we had to pool phloem samples after lyophilization by dates and diversity level to reach the minimum amount of material required for isotope analyses.

### 2.6 CO_2_ sample for isotopic analyses

C‐isotopic signatures of CO_2_ respired by leaves and soil CO_2_ efflux were calculated using a Keeling plot approach. Briefly, this is a two end‐member mixing model between the CO_2_ emitted by a source and background atmospheric CO_2_, with which the isotopic signature of the source can be determined (Keeling, 1958). The Keeling‐plot approach relies on the assumption that only two components are mixed together. This assumption is easily verified for leaf respiration, but soil CO_2_ efflux results from both belowground autotrophic respiration and heterotrophic respiration. However, it is possible to gather them as one soil respiratory flux if they are well mixed and their relative contributions to the overall flux remain constant over the sampling period (Pataki et al., 2003). Our sampling method insured that such combinations of sources could be made for soil respiration. Only Keeling plots with a *R* ^2^ > 0.95 were considered valid and used for further analyses.

For C‐isotopic signatures of soil CO_2_ efflux measurements the same chamber as for soil CO_2_ efflux was used (see above). For C‐isotopic signatures of leaf respiration measurements a custom‐made PVC chamber (15 × 7 × 4 cm) equipped with a septum was used. The chamber was connected to the closed‐path infrared gas analyser of the Li‐840 to monitor chamber [CO_2_]. The mixing model was based on three samples of chamber air collected at regular intervals over a [CO_2_] increase in at least 100 μmol/mol and injected in vials (12 ml, Exetainer^®^, Labco Ltd, High Wycombe, UK) that had been previously evacuated (<4.10^3^ Pa) and filled with N_2_. All leaf respiration samples were collected on leaves shaded the night before to avoid light‐enhanced dark respiration (Barbour et al., 2007). Vials were stored in a CO_2_‐free environment and δ^13^C measured within a week.

### 2.7 Water sample for isotopic analyses

Leaves sampled for water‐^18^O analyses were transferred in glass tubes and immediately frozen in liquid N_2_. Bark was removed from twigs sampled for xylem‐^18^O analyses, then promptly transferred in glass tubes and immediately frozen in liquid N_2_. Water was extracted from the leaves and twigs by cryogenic vacuum distillation as described in Barnard et al. (2007). Briefly, the tubes containing the frozen plant material were placed in an 80°C water bath connected to a vacuum system (ca. 4.10^−2^ mbar) including water traps that were cooled with liquid N_2_. The water was then transferred into 2‐ml vials and kept frozen until δ^18^O analysis (see below). Because of the small size of the sampled twigs extracted xylem water samples were pooled together by diversity level and sampling dates to have enough material to allow isotopic measurements.

### 2.8 C‐isotopes measurements

δ^13^C values of plant biomass, soil and phloem organic matter were measured with an elemental analyser (EA 1110 Series, Carlo Erba, Rhodano, Italy) coupled to an isotope‐ratio mass spectrometer (Delta S, Finnigan MAT, Bremen, Germany). The long‐term precision (~1.5 years) of the laboratory quality control standard (Catalpa leaf) was 0.09‰. The δ^13^C values of gas samples were measured with a modified Gasbench II peripheral equipped with a custom‐built cold trap coupled to the isotope‐ratio mass spectrometer Delta^Plus^ XL (both components Thermo Finnigan, Bremen Germany). C‐isotopic composition was expressed as the relative difference of the isotope abundance ratio of a sample relative to that of the Vienna Pee Dee Belemnite (VPDB) international standard. This difference is expressed in per mil and defined as:

(1) $$\delta^{13}C=\left[\frac{{\left({}^{13}C{/}^{12}C\right)}_{\mathrm{sample}}}{{\left({}^{13}C{/}^{12}C\right)}_{\mathrm{VPDB}}}-1\right].$$

For clarity, we use the following notation depending on whether the product is (a) the bulk δ^13^C value of a given plant part (δ^13^C_p_: δ^13^C_leaf_, δ^13^C_phloem_); or (b) the δ^13^C value of respired CO_2_ (δ^13^C_R_: δ^13^C_Rleaf_, δ^13^C_Rsoil_). Respiratory isotopic fractionations between both above‐ and below ground‐respired CO_2_ and phloem used as a proxy for the C source fuelling the respiration were also calculated (see Supporting Information Appendices S1 and S2).

### 2.9 O‐isotopes measurements

δ^18^O values in xylem sap (δ^18^O_xylem‐water_), and leaf water (δ^18^O_leaf‐water_) samples were measured using a TC/EA (high temperature conversion/elemental analyser, ThermoFinnigan, Bremen, Germany) coupled with a DeltaPlus XP mass spectrometer according to Gehre, Geilmann, Richter, Werner, and Brand (2004). The precision was 0.12‰. O‐isotopic composition was expressed as the relative difference of the isotope abundance ratio of a sample relative to that of the international Vienna Standard Mean Ocean Water (VSMOW). This difference is expressed in per mil and defined as:

(2) $$\delta^{18}O=\left[\frac{{\left({}^{18}O{/}^{16}O\right)}_{\mathrm{sample}}}{{\left({}^{18}O{/}^{16}O\right)}_{\mathrm{VSMOW}}}-1\right].$$

Additional measurements of bulk leaf organic matter, atmospheric water vapor and rain samples for δ^18^O analyses are described in Supporting Information Appendix S1 and the results presented in Supporting Information Appendix S2.

### 2.10 C‐isotope discrimination

C‐isotope discrimination during photosynthesis was derived from nonisotopic gas‐exchange measurements. First, based on the widely accepted simplified model developed by Farquhar, O'Leary, and Berry (1982) and further referred to as derived photosynthetic C‐isotope discrimination (Δ_*i*_):

(3) $$\Delta_{i}=a+\left(b-a\right)\frac{c_{i}}{c_{a}},$$

where *a* is the fractionation occurring during CO_2_ diffusion in air through the stomatal pore (*a* = 4.4‰, Craig, 1954), *b* is the net fractionation caused by carboxylation, *c*_*a*_ and *c*_*i*_ are ambient and substomatal concentrations of CO_2_, respectively. For higher C_3_ plants, *b* mostly results from the fixation of CO_2_ by Rubisco, the carboxylation enzyme, estimated at *b*’ = 29‰ in spinach (Roeske & O'Leary, 1984) and some PEP‐carboxylase fixation, leading to an estimated value for *b* of 27‰ in ecological studies (Farquhar & Richards, 1984; Lloyd & Farquhar, 1994).

Additionally, C‐isotope discrimination during photosynthesis was also derived based on the extended model including the effect of mesophyll conductance (*g*_*m*_) and photorespiration as two additional processes susceptible to influence photosynthetic C discrimination. Respiratory fractionation (Δ_Rsubstrate‐product_ is estimated as fractionation between the δ^13^C value of respired CO_2_ by a given plot component X, that is, leaf‐ or soil‐respired CO_2_, and the δ^13^C value of its putative substrate) was also calculated (Details are provided in Supporting Information Appendix S1).

### 2.11 O‐isotope discrimination

We also calculated the enrichment in ^18^O relative to that of the source water:

(4) $$\Delta^{18}O_{\mathrm{leaf}-\mathrm{water}}=\frac{\delta^{18}O_{\mathrm{leaf}-\mathrm{water}}-\delta^{18}O_{\mathrm{xylem}-\mathrm{water}}}{1+\delta^{18}O_{\mathrm{xylem}-\mathrm{water}}},$$

where the xylem sap is considered as the source water.

### 2.12 Statistical analysis

Data were analyzed using R 3.1.1 (R Core Team, 2014). Leaf gas‐exchange variables were tested using ANCOVA with species diversity and day as main factors. Vapor pressure deficit (VPD) in the gas‐exchange cuvette was included as a covariate. For leaf dark respiration, leaf temperature, measured by the LI6400, was used as a covariate. δ^13^C values were tested using ANOVA with species diversity and day as main factors. Correlation between variables across treatments and day of measurement were tested using a linear regression. Where they are of no special interest effects of covariates and environmental variables are only presented in Supporting Information Table S1 and are not further discussed. Considering the limited number of replicates for which the time‐consuming and in part costly measurements could be taken and the large variation typical of a field experiment only large effects could be detected with strict significance levels of *p* < 0.05. In order to avoid missing important medium‐sized effects and to reduce the corresponding type‐II error rates we also present and discuss marginally significant effects with *p* < 0.1 (Cohen, 2013; Toft & Shea, 1983).

## 3 RESULTS

### 3.1 Ecophysiological variables and soil CO_2_ efflux

**Figure 3:**
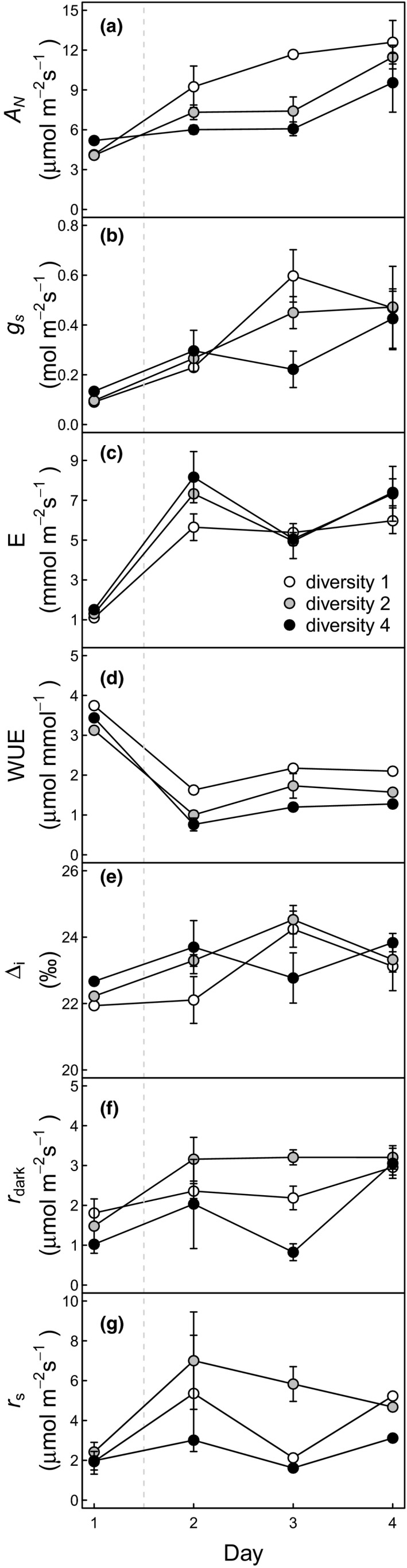
Response of leaf gas‐exchange variables of *Lithocarpus glaber* and soil CO_2_ efflux after a precipitation event in plots with different diversity: monoculture (diversity 1), two‐species mixture (diversity 2) and four‐species mixture (diversity 4). Measured variables are Net CO_2_ assimilation (*A*_*N*_, μmol m^−2^ s^−1^, panel a), stomatal conductance (*g*_*s*_, mol m^−2^ s^−1^, panel b), transpiration (*E*, mmol m^−2^ s^−1^, panel c), water‐use efficiency calculated from leaf gas‐exchange (WUE, μmol/mmol, panel d), the simplified prediction of photosynthetic C‐isotope discrimination (Δ_*i*_, ‰, panel e), leaf dark respiration (*r*_dark_, μmol m^−2^ s^−1^, panel f) and soil CO_2_ efflux (*r*_s_, μmol m^−2^ s^−1^, panel g). The dashed line represents the rain event that took place between day 1 and 2. Each point represents the average value (*n* ≥ 3) for a given diversity level on a given day. Error bars indicate ±1*SE*

CO_2_ assimilation (*A*_*N*_, *p* < 0.001, Figure 3a), stomatal conductance (*g*_*s*_, *p* < 0.001, Figure 3b) and transpiration (*E*,* p* < 0.001, Figure 3c) of the phytometer plants strongly increased after the rain. These responses to rain (i.e., increase in water availability) are in line with other studies (e.g., Delucia & Heckathorn, 1989; Lambers, Chapin, & Pons, 2008; Ponton, Dupouey, Breda, & Dreyer, 2002; Tambussi, Bort, & Araus, 2007). They are expected as higher water availability allows for higher *g*_*s*_ along with higher water loss through *E*. Thus, plants can open their stomata after the rain allowing for higher assimilation.

*A*_*N*_ of plants of the phytometer species was significantly higher in monoculture than in mixture (*p* = 0.022), especially during the first 2 days following the rain (day 2 and 3). After the rain, diversity had a marginally positive effect on *g*_*s*_ (*p* = 0.085) and on day 2, *E* was marginally lower in plants growing in monoculture than in those growing in mixture (*p* = 0.095).

After the rain, for all diversity levels, stronger increases in E (5–9 folds) relative to increases in *A*_*N*_ (3–4 folds) led to a significant decrease in water‐use efficiency (WUE; *p* < 0.001). A diversity effect on WUE was most notable after the rain event, with plants of the phytometer species in mixed‐species communities having lower WUE (*p* < 0.001, Figure 3d), the difference was driven by both higher *A*_*N*_ and lower E in monoculture compared to mixture, except on day 3 during which the difference mostly resulted from higher *A*_*N*_.

Derived photosynthetic C‐isotope discrimination (Δ_*i*_) of the phytometer plants changed significantly during the course of the experiment (*p* < 0.001, Figure 3e) with a general increase after the rain from day 1 to day 3. Species diversity had a marginal effect on Δ_*i*_ (*p* = 0.064). Δ_*i*_ was negatively related to VPD (*R* ^2^ = 0.28, *p* < 0.001). The extended model of photosynthetic discrimination incorporating the effect of mesophyll conductance and photorespiration showed similar responses to biotic and abiotic environment as Δ_*i*_, although it led to an increase in derived photosynthetic C‐isotope discrimination by a bit more than 1‰ (see details in Supporting Information Appendix S2). Importantly for our experiment, photosynthetic discrimination derived from gas‐exchange measurements (both with the simplified and more complex models) increased after the rain in agreements with numerous earlier studies linking changes between WUE and Δ_*i*_ at least within monocultures (e.g., Farquhar & Richards, 1984; Farquhar et al., 1989; Seibt, Rajabi, Griffiths, & Berry, 2008). These changes in Δ_*i*_ were sufficiently large to imprint new photoassimilates and thus allow tracing C in the plants through temporal changes in isotope values.

Leaf dark respiration (*r*_dark_) of phytometer plants did not change during our measurements, across species diversity and leaf temperature (Figure 3f). As expected, *r*_dark_ was positively correlated to *A*_*N*_ over the whole measurement period (*R* ^2^ = 0.27, *p* < 0.001) because with more C available more growth is possible and thus higher respiration results from increased growth (Lambers et al., 2008). Additionally, after the rain, species diversity had a significant effect on leaf respiration (*p* = 0.0043), with highest respiration rates of the target species in two‐species plots and lowest respiration rate in four‐species plots. Soil CO_2_ efflux near phytometer plants was significantly affected by species diversity (*p* = 0.005) with highest rates in two‐species mixtures and lowest rates in four‐species mixtures. Soil CO_2_ efflux was positively related to leaf respiration (*R* ^2^ = 0.41, *p* < 0.001).

### 3.2 δ^13^C of leaf‐respired CO_2_

**Figure 4:**
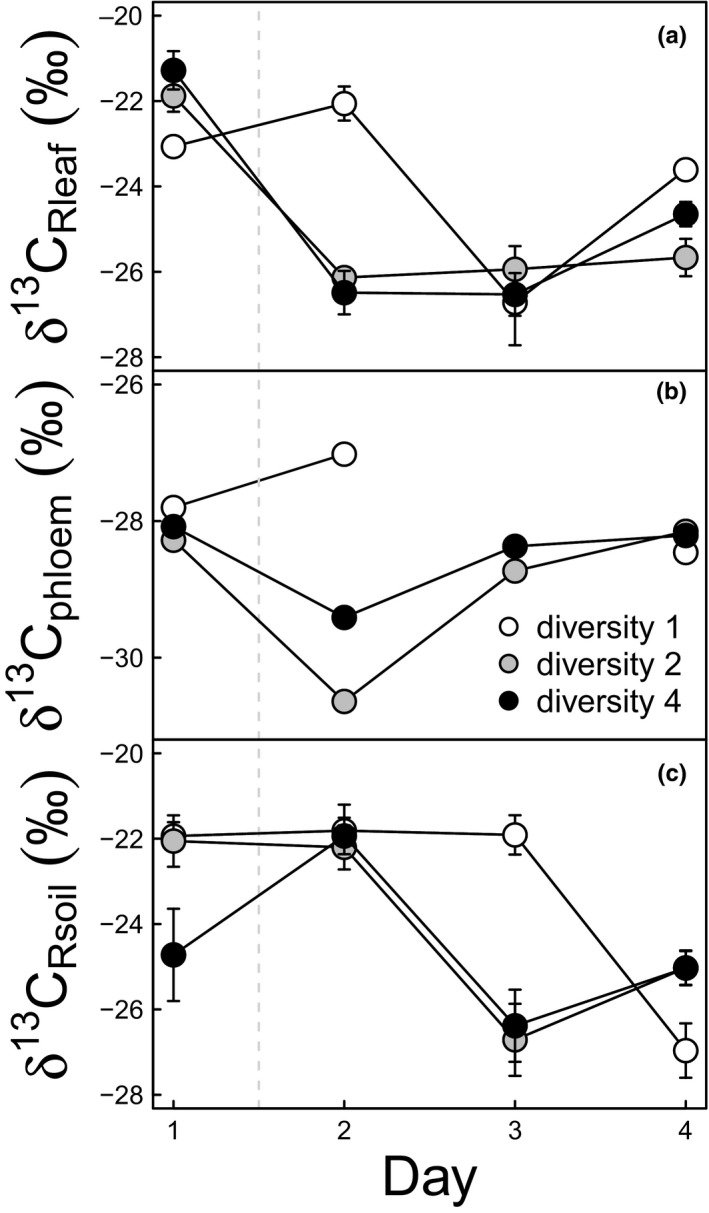
Response of δ^13^C values of leaf‐respired CO_2_ (δ^13^ C_R__leaf_, ‰, panel a), phloem organic matter (δ^13^C_phloem_, ‰, panel b) and soil‐respired CO_2_ (δ^13^ C_R__soil_, ‰, panel c) of *Lithocarpus glaber* to a precipitation event in plots with different diversity: monoculture (diversity 1), two‐species mixture (diversity 2) and four‐species mixture (diversity 4). The dashed line represents the rain event that took place between day 1 and 2. Each point represents the average value (*n* ≥ 3) for a given diversity level on a given day, except for phloem (*n* = 1). Error bars indicate ±1 *SE*

Both isotopic signatures of leaf‐ and soil‐respired CO_2_ (δ^13^C_Rleaf_ and δ^13^C_Rsoil_, respectively) decreased after the rain event (*p* < 0.001 for both, Figure 4a,c). Interestingly, the δ^13^C_Rleaf_ of phytometer plants in monoculture responded with a time lag (i.e., on day 3) to the water pulse whereas in mixed plots, δ^13^C_Rleaf_ decreased immediately after the night rain (i.e., on day 2).

The drop in δ^13^C_Rsoil_ occurred 1 day later than in δ^13^C_Rleaf_. In mixed plots, δ^13^C_Rsoil_ near phytometer plants decreased between day 2 and day 3 while in monoculture the decrease took place between day 3 and day 4. δ^13^C_Rleaf_ of phytometer plants was significantly higher between monocultures and mixtures (*p* = 0.027) due to the aforementioned time lag in monoculture. δ^13^C_Rleaf_ was positively related to WUE (*R* ^2^ = 0.39, *p* < 0.001).

### 3.3 Phloem isotopic signature

The dynamics of the response of bulk δ^13^C of phloem organic matter (δ^13^C_phloem_) of the phytometer plants to species diversity and time closely matched those of δ^13^C_Rleaf_ (correlation between the two measures *R* ^2^ = 0.30, *p* < 0.0001, Figure 4b), albeit more negative by several per mil difference. In monoculture, δ^13^C_phloem_ became slightly enriched (0.8‰) on day 2 following the rain event before decreasing. In mixtures, δ^13^C_phloem_ of the phytometer plants in contrast dropped after the rain and then increased in the next days. No ANOVA tests could be made for this observation because samples had to be pooled across replicates to obtain the measurements (see Materials and Methods section). δ^13^C_phloem_ was positively related to WUE (*R* ^2^ = 0.16, *p* = 0.022).

### 3.4 δ^18^O of leaf water and xylem water

**Figure 5:**
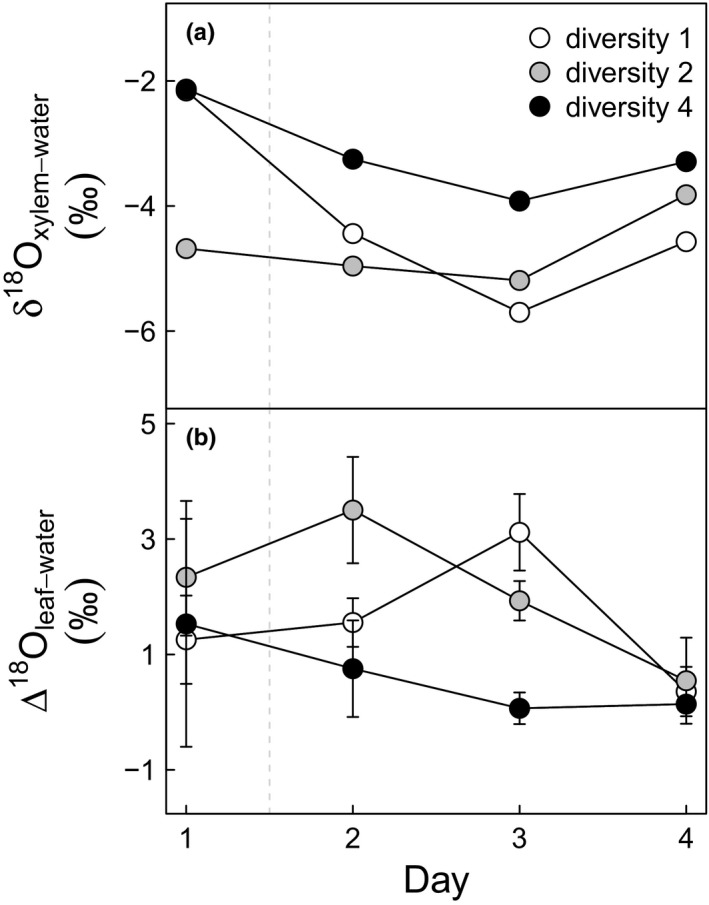
Response of xylem‐water δ^18^O (‰, panel a) and leaf‐water Δ^18^O (‰, panel b) of *Lithocarpus glaber* to a precipitation event in plots with different diversity: monoculture (diversity 1), two‐species mixture (diversity 2) and four‐species mixture (diversity 4). The dashed line represents the rain event that took place between day 1 and 2. Each point represents the average value (*n* ≥ 3) for a given diversity level on a given day. Error bars indicate ±1 *SE* (there is no error bar for xylem water δ^18^O, see text)

δ^18^O_leaf‐water_ of the phytometer plants significantly decreased over time (*p* = 0.028, Supporting Information Figure S4). δ^18^O_leaf‐water_ was positively related to WUE (*R* ^2^ = 0.13, *p* = 0.039). Despite the lack of power due to sample pooling, δ^18^O_xylem‐water_ was marginally affected by sampling dates (*p* < 0.095), decreasing after the rain before increasing on day 4, and by species diversity (*p* = 0.073, Figure 5a, no error bar, see above). Δ^18^O_leaf‐water_ was significantly affected by diversity (*p* = 0.039, Figure 5b) with phytometer plants growing in four‐species mixtures showing an immediate decline after the rain, while phytometer plants in two‐species mixtures had increased values for 1 day after the rain before the values decreased again on the third day. Phytometer plants in monocultures responded with a similar pattern, but 1 day later.

## 4 DISCUSSION

Our results highlight an effect of plot species richness on C dynamics in young phytometer plants of *L. glaber*. All phytometer plants reacted to the rain and the increased water availability by increasing assimilation and decreasing water‐use efficiency. However, plants in monocultures had a slower C transfer from assimilation to respiration and higher water‐use efficiency despite equal micro‐environmental conditions, suggesting that under intraspecific competition seedlings had lower water availability than seedlings under interspecific competition. Furthermore, the higher assimilation rate in plants growing in monocultures did not appear to translate into faster growth (Baruffol, 2014; see data in Supporting Information Table S2).

### 4.1 Increasing species diversity decreases competition for soil water

Slower C transfer to both above‐ and belowground respiration of and near phytometer plants in monoculture (Figure 4) suggests that these plants are more stressed than those growing in mixed plots, despite higher CO_2_ assimilation rate (but see below). Indeed, the 1‐day delay of the isotopic signal‐change following the rain event in δ^13^C of leaf‐respired CO_2_, δ^13^C phloem and δ^13^C of soil‐respired CO_2_ indicated a slower C turnover which has been associated with higher stress and particularly low water availability in plants (Ruehr et al., 2009). This is also in agreement with the Münch hypothesis of phloem transport (further details about the role of phloem transport in the observed response are also presented in Supporting Information Appendix S3). The decline in water availability increases the competition for water between transpiration and phloem transport, resulting in more viscous sap that moves slower (Hölttä et al., 2005; Lacointe & Minchin, 2008), as observed in our monoculture plants.

Our hypothesis of higher drought stress of the phytometer plants in monoculture than in species mixtures is further supported by the following results. First, it is supported by the observed higher WUE of phytometer plants in monoculture than in mixed‐species plots. Increased WUE is usually observed in plants facing moderate water shortage as a result of a proportionally stronger decline in water loss (transpiration) than in C assimilation (e.g., Lambers et al., 2008; Martin & Ruiz‐Torres, 1992; Quick et al., 1992). Second, leaf water enrichment (Δ^18^O_leaf‐water_ ≈ δ^18^O_leaf‐water_ − δ^18^O_xylem‐water_) of phytometer plants in monoculture and two‐species mixtures increased after the rain event and the resulting changes in humidity, indicating that the observed increased transpirative water loss is not immediately compensated by increased water uptake (Farquhar, Cernusak, & Barnes, 2007). In contrast, phytometer plants in four‐species mixtures showed little changes in Δ^18^O_leaf‐water_, suggesting that the increase in transpiration following the rain was rapidly replenished by higher water uptake. In contrast, Wang, Yakir, and Avishai (1998) found a negative correlation between δ^18^O_leaf‐water_ and WUE across species under different sampling conditions. Wang et al. (1998) compared species under stable environmental conditions, while the current study focuses on the eco‐physiological response of one species to dynamic changes in environmental conditions. Whereas a recent study (Trogisch, Salmon, He, Hector, & Scherer‐Lorenzen, 2016), conducted in the same experiment with another set of species, *Castanea henryi*,* Quercus serrata, Elaeocarpus decipiens* and *Schima superba,* suggests that niche differentiation for water resources may not yet be established for these young trees, our results show that their physiology is already responding to differences in the biotic environment presenting intra‐ vs. interspecific competition and various diversity backgrounds. Overall, the observed temporal dynamics of WUE, Δ^18^O_leaf‐water_ and δ^18^O_xylem‐water_ support the hypothesis that increased diversity in dense stands of young trees can reduce drought stress in phytometer plants of the selected target species *L. glaber*.

### 4.2 Effects of species diversity on C balance and growth of plants

Higher C assimilation and intermediate dark respiration should have favoured the growth of phytometer plants in monoculture compared to two‐species mixture, where plants had the highest dark respiration and lower assimilation. Phytometer plants in four‐species mixture having intermediate assimilation and the lowest respiration were expected to have intermediate growth. However, these expected results do not match aboveground‐biomass data (Baruffol, 2014; p. 84, Supporting Information Table S2): on average *L. glaber* plants grew largest in two‐species mixture (31.1 g per individual) and smallest in four‐species mixture (19.7 g), with monoculture plants being intermediate (23.2 g). To explain the results above, it should be noted that C loss results not only from respiration, but also from exudation. While our experiment does not allow to pinpoint the underlying mechanisms for the discrepancy between gas‐exchange and growth, it strongly suggests the existence of a nongrowth‐related C sink in monoculture, such as C storage, exudation favouring mycorhizal interaction (Walker, Bais, Grotewold, & Vivanco, 2003) or investment in secondary compound such as defense mechanisms both above and below ground. The latter is consistent with the increased predation and pathogen pressure on seeds observed in monoculture (Dalling, Davis, Schutte, & Elizabeth Arnold, 2011), with results showing that overyielding in species‐rich plots could result from higher root pathogen pressure in monoculture (De Kroon et al., 2012), and with less negative soil‐feedbacks in grassland species selected in monoculture as opposed to mixtures (Zuppinger‐Dingley, Flynn, de Deyn, Petermann, & Schmid, 2016).

The absence of a significant respiration response to rain is in agreement with previous studies because leaf respiration is less sensitive to dry conditions than photosynthesis, even under intense stress (Schwalm et al., 2010). A review showed that respiration in about one‐third of the studied species was insensitive to drought while the other two‐third showed a decrease in respiration with decreasing water availability (Atkin & Macherel, 2009). This heterogeneity of respiratory response to water resources has been explained by the complex regulation between available nonstructural carbohydrates and the up‐ or down‐regulation of processes with high C‐respiratory cost, for example, protein synthesis, turnover and growth (Gibon et al., 2009; Hummel et al., 2010).

## 5 CONCLUSION

The establishment phase plays a critical role in shaping tree communities because it can influence stand development in the longer term (Kobe, 1996) and it directly controls the pool of species that will survive and compose the mature forest (Baraloto, Goldberg, & Bonal, 2005). A better understanding of processes regulating biotic interactions in dense young tree communities is crucial to predict the future of forests. Overall, our results show that young trees of a phytometer species have slower C dynamics when grown in monoculture than in mixtures. At this early stage, in which competition for light is still weak, this slower response appears to be driven by lower water availability in monoculture than in mixtures. These positive effects of species diversity on C dynamics may have counterbalanced negative effects on assimilation rates for phytometer plants and as a consequence higher assimilation rates in monoculture may not have led to increased growth and biomass.

## CONFLICT OF INTEREST

None declared.

## AUTHOR CONTRIBUTION

YS, MK, RTS and BS conceived the ideas and designed methodology; YS and BY collected the data; YS and XL analyzed the data; YS and XL led the writing of the manuscript. All authors contributed critically to the drafts and gave final approval for publication.
