# Supplementary material for: Surrounding species diversity improves subtropical seedlings’ carbon dynamics
Source: Ecol Evol. 2018 Jun 22;8(14):7055–67. doi: 10.1002/ece3.4225 (PMC6065279; doi:10.1002/ece3.4225)
Supplement: Supplementary file 1 [file ECE3-8-7055-s001.docx]

**Supporting Information**

- Appendix S1: Additional method
- Appendix S2: Additional results
- Appendix S3: Additional discussion
- Appendix S4: Additional figures and tables
- Appendix S5: Supporting references

**Appendix S1: Additional method**

**1.1 Additional δ^13^C measurements: microbial biomass and atmospheric CO_2_**

Prior to extraction, samples for soil microbial biomass measurements were stored at 4 °C. Soil microbial biomass was extracted by fumigation-extraction after soil sieving (2 mm mesh, Vance et al., 1987, Wu et al., 1990). For each soil sample, one 10-g subsample was fumigated 24 h with chloroform vapour before extracting microbial biomass, while a second 10-g subsample was extracted without prior fumigation. Microbial C was extracted by vigorous shaking for 30 min in 30 mM K_2_SO_4_ solution, then the extracts were filtered, frozen (–80°C) and lyophilised before isotope-composition and C-concentration analysis.

δ^13^C values of soil microbial biomass (δ^13^C_mic_) was calculated using the following mass balance equation:

|  | (S1) |
| --- | --- |

where δ^13^C_F_ and δ^13^C_NF_ are the δ^13^C values measured (see main document) in fumigated and non-fumigated subsamples, respectively, and C_F_ and C_NF_ are C concentrations in fumigated and non-fumigated subsamples, respectively.

Measurements of background δ^13^C of atmospheric CO_2_ were done to account for potential changes in δ^13^C value of background CO_2_. We measured it both directly several times during the course of the experiment and integrated over the growing season using well-watered *Zea mays* L. plants because C_4_ plants show fairly constant C discrimination under non-limiting conditions (Evans et al., 1986, Buchmann et al., 1996). At the time of the experiment *Zea mays* leaf biomass was sampled and processed for δ^13^C analysis as described in the main document. δ^13^C of atmospheric CO_2_ was stable during the course of the experiment with an average value of –9.34±0.25 ‰ and thus data did not need to be corrected for changes in background δ^13^C of atmospheric CO_2_.

**1.2 Photosynthetic discrimination extended model**

In addition to the simplified model for photosynthetic discrimination presented in the manuscript, C isotope discrimination during photosynthesis was also derived based on the extended model including the effect of mesophyll conductance (g_m_) and photorespiration as two additional processes susceptible to influence photosynthetic C discrimination (Farquhar et al., 1989).

| $\Delta_{ic}=a\frac{\left( C_{a}-C_{i} \right)}{C_{a}}+a_{m}\frac{\left( C_{i}-C_{c} \right)}{C_{a}}+b'\frac{C_{c}}{C_{a}}-f\frac{\Gamma_{*}}{C_{a}}$ | (S2) |
| --- | --- |

where *a*_m_ is the fractionation during CO_2_ transfer from intercellular air spaces to the chloroplast (1.8‰; Farquhar et al., 1989), c_c_ is the partial pressures of CO_2_ at the carboxylation site, *f* is fractionation during photorespiration (16.2‰; Evans and Von Caemmerer, 2013), *Γ_*_* is the CO_2_ compensation point in the absence of dark respiration (calculated according to Brooks and Farquhar, 1985), from the temperature response: *Γ*_*_ = 42.7 + 1.68 (*t*-25) + 0.0012 (*t*-25)^2^, where *t* is temperature in °C and *Γ*_*_ is expressed in µmol mol^-1^). c_c_ was calculated from A=(c_i_-c_c_)g_m_/P where P is the atmospheric pressure and A the CO_2_ assimilation rate (Harley et al., 1992). Following Flanagan and Farquhar (2014) we used two scenarios for g_m_, with either g_m_=1.5g_s_ and g_m_=3g_s_ (Warren and Dreyer, 2006, Warren et al., 2003, Seibt et al., 2008) leading to two scenarios for Δ_ic_, noted Δ_ic1.5_ and Δ_ic3_, respectively. Note that the effects of day respiration were not included in the equation following Flanagan & Farquhar (2014), since it is supposed minimal. Photoassimilate isotopic signature was calculated from equations 5 or 6 and equation 3 using background atmospheric CO_2_ isotopic values for δ_source_.

**1.3 Respiratory fractionation**

C isotope discrimination during any biochemical reaction (i.e. from a source to a product) can be calculated according to Farquhar, O'Leary & Berry (1982):

|  | (S3) |
| --- | --- |

where Δ is the discrimination between δ_source_ and δ_product_, the δ^13^C values of the source and of the product of the reaction, respectively. Unlike δ values, discrimination is independent of the isotopic composition of the source. In particular, respiratory fractionation (Δ_Rsubstrate-product_) is estimated as discrimination between the δ^13^C value of CO_2_ respired by a given plot component (X, leaf- or soil-respired CO_2_) and the δ^13^C value of its putative substrate:

| $\Delta_{Rsubstrate-product}=\frac{\delta_{S}-\delta_{R-X}}{1+\delta_{R-X}}$ | (S4) |
| --- | --- |

where δ_s_ is the δ^13^C value of the putative substrate and δ_R-X_ is the δ^13^C value of the CO_2_ respired by X. We are aware that defining the exact substrate for soil respiration exceeded our experimental capabilities, making calculations of respiratory fractionation associated with soil CO_2_ challenging. Thus, we used phloem organic matter since phloem is the main source of C for non-autotrophic tissues, and it integrates ^13^C variation over periods of time similar to our sampling frequencies, i.e. hours. Note that Δ_Rphloem-leaf_ and Δ_Rphloem-soil_ were calculated from the isotopic signature of pooled phloem sample as described above.

**1.4** **Additional δ^18^O measurements: bulk leaf organic matter,** **atmospheric water vapour and rain samples for isotopic analyses**

Atmospheric water vapour was collected by cryogenic condensation (Barnard et al., 2007). Air was pumped from the atmosphere above the canopy at 35 l h^-1^ for at least two hours every day through a water trap submersed in ethanol cooled with liquid N_2_ to a temperature of ca. –70°C). The collected water was then transferred into 2-ml vials and kept frozen until δ^18^O analysis (see main document). Rain-water samples were contaminated and could not be used in our experiments. However, in accordance with the temporal isotopic precipitation pattern modelled for our field site, δ^18^O in precipitation was expected to be 7.9 ‰ (Bowen et al., 2005, Bowen, 2015).

For bulk leaf material 0.5 mg of homogenized sample dried at 60°C was transferred in silver capsules (Säntis Analytical AG, Teufen, Switzerland). The samples were combusted in a TC/EA coupled to an isotope ratio mass spectrometer (Delta^Plus^ XP; Finnigan MAT GmbH, Bremen, Germany). Otherwise, δ^18^O values in leaf organic matter (δ^18^O_leaf-om_) and atmospheric water-vapour (δ^18^O_atm-water_) samples were measured according to the protocol described in the main document.

**Appendix S2: Additional results**

**2.1 δ^13^C value of soil, leaf and microbial biomass**

Bulk δ^13^C of soil organic matter (δ^13^C_soil_) was -26.3±0.1 ‰ and was not affected by sampling date or diversity. Bulk δ^13^C of leaf biomass (δ^13^C_leaf_) was marginally affected by plot diversity (p=0.099) but did not change over time during the course of our experiment. The δ^13^C_leaf_ values of the target species growing in monoculture was –28.8±0.2 ‰, in two-species mixture it was –28.8±0.1 ‰ and in four-species mixture it was –29.1±0.2 ‰. The only marginal significance of the diversity effect is not so surprising because the δ^13^C of leaf organic-matter represents longer-term integration of C assimilation and the plants of the target species had been transferred from the nursery into the differently diverse experimental communities only a few months prior to the start of the experiment. Thus, only a fraction of the total leaf area was built in the field under the different diversity regimes. However, based on gas-exchange and phloem δ^13^C diversity effect are likely to become stronger over time.

Microbial biomass did not changed during the course of the experiment and was not affected by plant diversity with an average value of –27.8±0.2 ‰, possibly again due to the short time between planting of experimental communities and sampling. Furthermore the exported mass of C is so small relative to the already available soil-C that this change resulting from new C is by far below the detection limit. These results will thus not be further discussed.

**2.2 Photosynthetic discrimination extended model**

Incorporation of mesophyll conductance and photorespiration effect in the derived photosynthetic C isotope discrimination lead to an average decrease in discrimination by 1.28±0.16‰ between Δ_i_ and Δ_ic1.5_ and an average decrease in discrimination by 1.11±0.10‰ between Δ_i_ and Δ_ic3_. However, the responses of Δ_ic1.5_ and Δ_ic3_ to diversity and time after the rain event showed the same pattern as Δ_i_ (Fig. S2). Both Δ_ic1.5_ and Δ_ic3_ were significantly affected by the day of measurement (both p<0.001), but not by plot diversity. Additionally, a marginally significant interaction between monoculture versus mixture and day of measurement was observed for both Δ_ic1.5_ and Δ_ic3_ (p=0.057 and p=0.058, respectively). Δ_ic1,5_  and Δ_ic3_ were negatively related to VPD (R^2^=0.32, p<0.001 and R^2^=0.36, p<0.001, respectively)

**2.3 Respiratory fractionation**

Apparent respiratory fractionation was large both above- and belowground measured values for Δ_Rphloem-leaf_, and Δ_Rphloem-soil_ between 2 and 8‰ (Fig. S3). Δ_Rphloem-leaf_ was significantly affected by the sampling date (p<0.001). Similarly, Δ_Rphloem-soil_ was significantly affected by the day of sampling (p<0.001). Leaf temperature (measured by the Li6400) had no effect on respiratory fractionation, nor did the surrounding diversity. However, a significant interaction between day of measurement and diversity (p=0.007) was observed for Δ_Rphloem-leaf_ (Fig. S3).

**2.4 δ^18^O of leaf organic matter and leaf atmospheric water vapour**

Over the duration of the experiment and across diversity levels, δ^18^O of leaf organic matter was on average +21.89±0.2 ‰ and δ^18^O of atmospheric water vapour was –7.3±0.2 ‰. Plot diversity and sampling dates had no effect on leaf organic matter δ^18^O.

**Appendix S3: Additional discussion**

**Isotope signal from phloem C transfer and respiratory fractionation**

The fast transfer of newly assimilated C from above to below ground and its release back into the atmosphere through respiration is in agreement with previous studies showing rapid transfer of labelled C (naturally as in this study or experimentally using enriched isotope tracer, e.g., Kodama et al., 2008, Salmon et al., 2014) in non-stressed plants (see also Kuzyakov and Gavrichkova, 2010, for a review). The time scale of the measured isotopic response to the water pulse given by the rain event is consistent with phloem transport rates of newly assimilated C (Kuzyakov and Gavrichkova, 2010). Although faster responses involving for example pressure gradient waves (Mencuccini and Hölttä, 2010) could have occurred, they would have been too fast to be detected in our experimental setup and would likely only have played a minor role in the total δ^13^C response. Indeed, phloem transport seemed to be the main driver of changes in above- and belowground respired ^13^CO_2_, because changes in δ^13^C_phloem_ (~2 ‰) after the rain pulse were comparable to changes in Δ_i_. This result suggests that the net effects of g_m_ or post-carboxylation discrimination on δ^13^C of photo-assimilates were less important in these young trees.

However, after the rain event, changes in δ^13^C of respired CO_2_ were about twice as large (~4 ‰), both at leaf and soil level, as those on Δ_i_ and δ^13^C_phloem_ (~2 ‰). Thus, if about half of the respiratory isotopic plant response is driven by transport of new assimilates, additional isotope changes in respiration have to be explained by non-photosynthetic fractionation in respiratory physiology. Changes in the δ^13^C of respired CO_2_ compared to its substrate are referred to as apparent respiratory fractionation (Ghashghaie et al., 2003). Although identifying molecular and physiological respiratory processes involved in apparent respiratory fractionation exceeds the goal of our study by far, recent progress in understanding these processes helps to shed light on the large apparent respiratory fractionation (Δ_Rphloem-leaf_ and Δ_Rphloem-soil_) observed in our plants (2–6 ‰). Apparent fractionation during respiration has been reported to result from several not-mutually exclusive processes that might take place here (see Bowling et al., 2008, Brüggemann et al., 2011, Werner and Gessler, 2011, Werner et al., 2011 for reviews and more details): 1) changes in isotope signature of the substrate due to respiration of C assimilated under different environmental conditions or changes in the nature of the substrate since for example lipids are more ^13^C depleted than sucrose or starch; 2) changes in respiratory fractionation per se, due to changes in respiratory metabolic pathways and 3) changes in the balance of different fluxes, e.g., the relative contribution of root and microbial respiration to soil CO_2_ efflux. Despite, the existence of complex post-photosynthetic processes in the C physiological response of our plants to their biotic and abiotic environment, our results suggest that phloem transport is the main driver of C dynamic in the studied species *L. glaber*.

**Insights in ecological processes underlying the plot community response to species richness**

At the plot scale (Baruffol, 2014, p87, reported for information in Table S2), the highest aboveground biomass was found for the two-species mixture of *L. glaber* with *S.mukorossi*, followed by the four-species mixture. Plots with *L. glaber* and *S.mukorossi* produced ten times the biomass as the monoculture of *L. glaber*. Few studies have ever looked at gas-exchange responses to biodiversity, limiting our ability to understand the underlying processes. One study of temperate grassland showed that assimilation at the plot level increased with diversity (De Boeck et al., 2007), possibly as a result of *i*) an average increase in assimilation for all species or *ii*) a sampling effect due to the presence of some species having a high assimilation rate in mixtures. While studies of the response of soil CO_2_ efflux to biodiversity have rarely been conducted (van Haren et al., 2013), the existing experiments show that soil CO_2_ efflux depends more on species composition (Johnson et al. 2008) than on biodiversity *per* se (Cahill, 2003). Our results combined with the biomass data of Baruffol (2014, p114 and Table S2) suggest a strong selection effect in two species mixture in the studied community in line with process in De Boeck et al. (2007) and the conclusion of Johnson et al (2008). Moreover, recent evidence of effects of neighbour presence on root growth (Chen et al., 2015) and communication through common mycelial networks between host plants (see recent review by Johnson and Gilbert, 2015, see also Klein et al., 2016) suggest mechanisms for belowground competition that might have contributed to the observed effect.

**Appendix S4: Additional figures and tables**

**Figure S1:** leaf respiration over CO_2_ assimilation ratio, calculated from the averaged assimilation and leaf respiration values for each day and diversity level (Hence the lack of error bar)

**Figure S2:** temporal dynamic of photosynthetic discrimination (Δ_i_, panel A) and photosynthetic discrimination including mesophyll conductance (Δ_ic1,5_  and Δ_ic3_, panel B and C, respectively). Values are given as average per day and diversity level ± 1 S.E.

**Figure S3:** Response of apparent leaf- and soil-respiratory fractionation (Δ_Rphloem-leaf_, ‰, panel A and Δ_Rphloem-soil_, ‰, panel B, respectively) to a precipitation event in plots with different diversity: monoculture (diversity 1), two-species mixture (diversity 2) and four-species mixture (diversity 4). The dashed line represents the rain event that took place between day 1 and 2. Each point represents the average value (n≥3) for a given diversity level on a given day. Error bars indicate ±1SE.

**Figure S4:** Response of leaf-water δ^18^O (‰), to a precipitation event in plots with different diversity: monoculture (diversity 1), two-species mixture (diversity 2) and four-species mixture (diversity 4). The dashed line represents the rain event that took place between day 1 and 2. Each point represents the average value (n≥3) for a given diversity level on a given day. Error bars indicate ±1SE.

| Variables | relation with environmental variables | | | | |  | covariates | |
| --- | --- | --- | --- | --- | --- | --- | --- | --- |
|  | wind speed | air humidity | atmospheric pressure | air temperature | soil temperature (20cm) |  | VPD in gas-exchange cuvette | leaf temperature |
| A_N_ | ns | ns | - R^2^=0.44, p<0.001 | - R^2^=0.15, p=0.027 | ns |  | ns | na |
| g_s_ | - R^2^=0.17, p=0.017 | ns | - R^2^=0.17, p=0.016 | na | ns |  | p<0.001 | na |
| E | - R^2^=0.72, p<0.001 | + R^2^=0.16, p=0.023 | - R^2^=0.26, p=0.002 | - R^2^=0.40, p<0.001 | ns |  | p=0.008 | na |
| WUE | + R^2^=0.53, p<0.001 | ns | ns | + R^2^=0.20, p=0.008 | ns |  | p=0.038 | na |
| leaf respiration | ns | ns | ns | + R^2^=0.14, p=0.026 | ns |  | na | ns |
| δ^13^C_Rleaf_ | + R^2^=0.16, p=0.010 | ns | ns | ns | ns |  | na | na |
| δ^13^C_Rsoil_ | ns | + R^2^=0.25, p<0.001 | + R^2^=0.19, p=0.005 | ns | - R^2^=0.41, p<0.001 |  | na | na |
| Δ_Rleaf-phloem_ | ns | ns | ns | ns | ns |  | na | na |
| Δ_Rsoil-phloem_ | ns | + R^2^=0.49, p<0.001 | + R^2^=0.20, p=0.006 | - R^2^=0.19, p=0.007 | - R^2^=0.44, p<0.001 |  | na | na |

**Table S1:** relation between ecophysiological and isotopic variables with environmental variables, as well as the effect of covariates in our statistical model. + and – indicates a positive and negative correlation, respectively. na indicates that the test was not performed and ns indicates non-significant results. Variables with no covariates or no significant relation with the environmental variables are not reported here to avoid overly a complicated table.

|  | monoculture | 2-species mixture | 4-species mixture |
| --- | --- | --- | --- |
| *Lithocarpus glaber*  average biomass (g) | 23.24 | 31.09 | 19.68 |
| average plot biomass (g) | 228 | lg-cm: 105 | 1600 |
|  |  | lg-cs: 545 |  |
|  |  | lg-sm: 2807 |  |

**Table S2:** Biomass data for the studied plot and species from Baruffol (2014, p84 and 87). Species names are abbreviated as: lg: *Lithocarpus glaber*, cs: *Castanopsis sclerophylla*, cm: *Cyclobalanopsis myrsinaefolia* and sm: *Sapindus mukorossi*.

**Appendix S5: Supporting references**

BARNARD, R. L., SALMON, Y., KODAMA, N., SORGEL, K., HOLST, J., RENNENBERG, H., GESSLER, A. & BUCHMANN, N. 2007. Evaporative enrichment and time lags between δ^18^O of leaf water and organic pools in a pine stand. *Plant Cell And Environment,* 30**,** 539-550.

BARUFFOL, M. 2014. *Aboveground primary productivity in forest ecosystems as a function of species richness and composition.* Dr. sc. nat., Universität Zürich.

BOWEN, G., WASSENAAR, L. & HOBSON, K. 2005. Global application of stable hydrogen and oxygen isotopes to wildlife forensics. *Oecologia,* 143**,** 337-348.

BOWEN, G. J. 2015. *The Online Isotopes in Precipitation Calculator, version 2.2* [Online]. Available: <http://www.waterisotopes.org>. [Accessed].

BOWLING, D. R., PATAKI, D. E. & RANDERSON, J. T. 2008. Carbon isotopes in terrestrial ecosystem pools and CO_2_ fluxes *New Phytologist,* 178**,** 24-40.

BROOKS, A. & FARQUHAR, G. D. 1985. Effect of temperature on the CO_2_/O_2_ specificity of ribulose-1,5-bisphosphate carboxylase/oxygenase and the rate of respiration in the light. *Planta,* 165**,** 397-406.

BRÜGGEMANN, N., GESSLER, A., KAYLER, Z., KEEL, S. G., BADECK, F., BARTHEL, M., BOECKX, P., BUCHMANN, N., BRUGNOLI, E., ESPERSCHÜTZ, J., GAVRICHKOVA, O., GHASHGHAIE, J., GOMEZ-CASANOVAS, N., KEITEL, C., KNOHL, A., KUPTZ, D., PALACIO, S., SALMON, Y., UCHIDA, Y. & BAHN, M. 2011. Carbon allocation and carbon isotope fluxes in the plant-soil-atmosphere continuum: a review. *Biogeosciences,* 8**,** 3457-3489.

BUCHMANN, N., BROOKS, J. R., RAPP, K. D. & EHLERINGER, J. R. 1996. Carbon isotope composition of C_4_ grasses is influenced by light and water supply *Plant, Cell & Environment,* 19**,** 392-402.

CAHILL, J. F. 2003. Neighbourhood-scale diversity, composition and root crowding do not alter competition during drought in a native grassland. *Ecology Letters,* 6**,** 599-603.

CHEN, B. J. W., DURING, H. J., VERMEULEN, P. J., DE KROON, H., POORTER, H. & ANTEN, N. P. R. 2015. Corrections for rooting volume and plant size reveal negative effects of neighbour presence on root allocation in pea. *Functional Ecology,* 29**,** 1383-1391.

DE BOECK, H. J., LEMMENS, C., VICCA, S., VAN DEN BERGE, J., VAN DONGEN, S., JANSSENS, I. A., CEULEMANS, R. & NIJS, I. 2007. How do climate warming and species richness affect CO_2_ fluxes in experimental grasslands? *New Phytologist,* 175**,** 512-522.

EVANS, J. R., SHARKEY, T. D., BERRY, J. A. & FARQUHAR, G. D. 1986. Carbon isotope discrimination measured concurrently with gas-exchange to investigate CO_2_ diffusion in leaves of higher-plants. *Australian Journal of Plant Physiology,* 13**,** 281-292.

EVANS, J. R. & VON CAEMMERER, S. 2013. Temperature response of carbon isotope discrimination and mesophyll conductance in tobacco. *Plant, Cell & Environment,* 36**,** 745-756.

FARQUHAR, G. D., EHLERINGER, J. R. & HUBICK, K. T. 1989. Carbon isotope discrimination and photosynthesis. *Annual Review of Plant Physiology and Plant Molecular Biology,* 40**,** 503-537.

FARQUHAR, G. D., O'LEARY, M. H. & BERRY, J. A. 1982. On the relationship between carbon isotope discrimination and the intercellular carbon dioxide concentration in leaves. *Australian Journal of Plant Physiology,* 9**,** 121-137.

FLANAGAN, L. B. & FARQUHAR, G. D. 2014. Variation in the carbon and oxygen isotope composition of plant biomass and its relationship to water-use efficiency at the leaf- and ecosystem-scales in a northern Great Plains grassland. *Plant Cell and Environment,* 37**,** 425-438.

GHASHGHAIE, J., BADECK, F.-W., LANIGAN, G., NOGUÉS, S., TCHERKEZ, G., DELÉENS, E., CORNIC, G. & GRIFFITHS, H. 2003. Carbon isotope fractionation during dark respiration and photorespiration in C_3_ plants. *Phytochemistry reviews,* 2**,** 145-161.

HARLEY, P. C., LORETO, F., DI MARCO, G. & SHARKEY, T. D. 1992. Theoretical considerations when estimating the mesophyll conductance to CO_2_ flux by analysis of the response of photosynthesis to CO_2_. *Plant Physiology,* 98**,** 1429-1436.

JOHNSON, D. & GILBERT, L. 2015. Interplant signalling through hyphal networks. *New Phytologist,* 205**,** 1448-1453.

KLEIN, T., SIEGWOLF, R. T. W. & KÖRNER, C. 2016. Belowground carbon trade among tall trees in a temperate forest. *Science,* 352**,** 342-344.

KODAMA, N., BARNARD, R. L., SALMON, Y., WESTON, C., FERRIO, J. P., HOLST, J., WERNER, R. A., SAURER, M., RENNENBERG, H., BUCHMANN, N. & GESSLER, A. 2008. Temporal dynamics of the carbon isotope composition in a *Pinus sylvestris* stand: from newly assimilated organic carbon to respired carbon dioxide. *Oecologia,* 156**,** 737-750.

KUZYAKOV, Y. & GAVRICHKOVA, O. 2010. Time lag between photosynthesis and carbon dioxide efflux from soil: a review of mechanisms and controls. *Global Change Biology,* 16**,** 3386-3406.

MENCUCCINI, M. & HÖLTTÄ, T. 2010. The significance of phloem transport for the speed with which canopy photosynthesis and belowground respiration are linked. *New Phytologist,* 185**,** 189-203.

SALMON, Y., BARNARD, R. L. & BUCHMANN, N. 2014. Physiological controls of the isotopic time lag between leaf assimilation and soil CO_2_ efflux. *Functional Plant Biology,* 41**,** 850-859.

SEIBT, U., RAJABI, A., GRIFFITHS, H. & BERRY, J. A. 2008. Carbon isotopes and water use efficiency: sense and sensitivity. *Oecologia,* 155**,** 441-454.

VAN HAREN, J., DE OLIVEIRA, R. C., BELDINI, P. T., DE CAMARGO, P. B., KELLER, M. & SALESKA, S. 2013. Tree species effects on soil properties and greenhouse gas fluxes in East-central Amazonia: comparison between monoculture and diverse forest. *Biotropica,* 45**,** 709-718.

VANCE, E. D., BROOKES, P. C. & JENKINSON, D. S. 1987. An extraction method for measuring soil microbial biomass C. *Soil Biology Biochemistry,* 19**,** 703-707.

WARREN, C. R. & DREYER, E. 2006. Temperature response of photosynthesis and internal conductance to CO_2_: results from two independent approaches. *Journal of Experimental Botany,* 57**,** 3057-3067.

WARREN, C. R., ETHIER, G. J., LIVINGSTON, N. J., GRANT, N. J., TURPIN, D. H., HARRISON, D. L. & BLACK, T. A. 2003. Transfer conductance in second growth Douglas-fir (*Pseudotsuga menziesii* (Mirb.)Franco) canopies. *Plant, Cell & Environment,* 26**,** 1215-1227.

WERNER, C. & GESSLER, A. 2011. Diel variations in the carbon isotope composition of respired CO_2_ and associated carbon sources: a review of dynamics and mechanisms. *Biogeosciences,* 8**,** 2437-2459.

WERNER, R. A., BUCHMANN, N., SIEGWOLF, R. T. W., KORNEXL, B. E. & GESSLER, A. 2011. Metabolic fluxes, carbon isotope fractionation and respiration – lessons to be learned from plant biochemistry. *New Phytologist,* 191**,** 10-15.

WU, J., JOERGENSEN, R. G., POMMERENING, B., CHAUSSOD, R. & BROOKES, P. C. 1990. Measurement of soil microbial biomass C by fumigation extraction - an automated procedure. *Soil Biology & Biochemistry,* 22**,** 1167-1169.
